# Direct Observation of Morphological and Chemical Changes during the Oxidation of Model Inorganic Ligand-Capped Particles

**DOI:** 10.1021/acsnano.4c08846

**Published:** 2024-12-19

**Authors:** Maximilian Jaugstetter, Xiao Qi, Emory M. Chan, Miquel Salmeron, Kevin R. Wilson, Slavomír Nemšák, Hendrik Bluhm

**Affiliations:** † Materials Sciences Division, Lawrence Berkeley National Laboratory, Berkeley, California 94720, United States; ‡ Molecular Foundry, Lawrence Berkeley National Laboratory, Berkeley, California 94720, United States; § Chemical Sciences Division, Lawrence Berkeley National Laboratory, Berkeley, California 94720, United States; ∥ Advanced Light Source, Lawrence Berkeley National Laboratory, Berkeley, California 94720, United States; ⊥ Department of Physics and Astronomy, University of California, Davis, California 95616, United States; # Fritz Haber Institute of the Max Planck Society, Berlin D-14195, Germany

**Keywords:** ambient pressure X-ray photoelectron spectroscopy, grazing incidence X-ray scattering, core–shell nanoparticles, oleic acid, oxidation

## Abstract

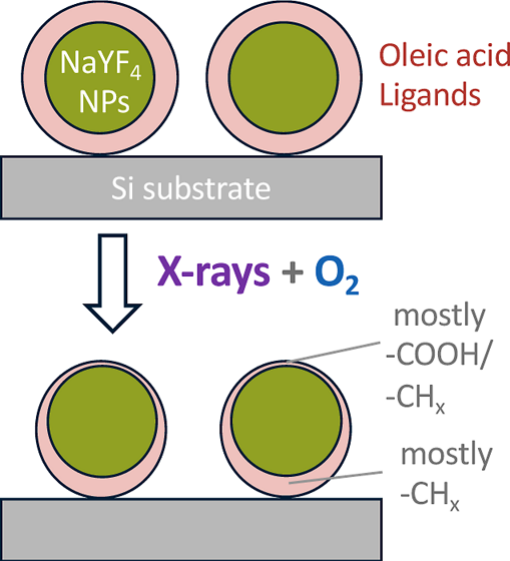

Functionalization and volatilization are competing reactions during the oxidation of carbonaceous materials and are important processes in many different areas of science and technology. Here, we present a combined ambient pressure X-ray photoelectron spectroscopy (APXPS) and grazing incidence X-ray scattering (GIXS) investigation of the oxidation of oleic acid ligands surrounding NaYF_4_ nanoparticles (NPs) deposited onto SiO_*x*_/Si substrates. While APXPS monitors the evolution of the oxidation products, GIXS provides insight into the morphology of the ligands and particles before and after the oxidation. Our investigation shows that the oxidation of the oleic acid ligands proceeds at O_2_ partial pressures of below 1 mbar in the presence of X-rays, with the oxidation eventually reaching a steady state in which mainly CH_*x*_ and –COOH functional groups are observed. The scattering data reveal that the oxidation and volatilization reaction proceeds preferentially on the side of the particle facing the gas phase, leading to the formation of a chemically and morphologically asymmetric ligand layer. This comprehensive picture of the oxidation process could be obtained only by combining the X-ray scattering and APXPS data. The investigation presented here lays the foundation for further studies of the stability of NP layers in the presence of reactive trace gases and ionizing radiation and for other nanoscale systems where chemical and morphological changes happen simultaneously and cannot be understood in isolation.

## 1 Introduction

Organic ligands are widely used as protective layers around nanoparticles (NPs) to prevent the particles from coagulation, to passivate surface charge traps, and to provide chemical stability.^1^ The efficacy of the ligand layer depends on the strength of the bonding of the ligand molecules to the NP, the ligand interaction with the surrounding medium (often an organic or aqueous solution), and its stability over time. The protective properties of the capping ligand might considerably change if their molecular structure is altered through chemical reactions. One example is the oxidation of unsaturated fatty acids,^2^ which are widely used as ligands, by ozone, which is a trace gas in the atmosphere and is also produced through the interaction of ionizing radiation with, for instance, oxygen and water vapor. For the understanding of the stability or, adversely, the aging of NP–ligand bonds under realistic environmental settings, e.g., sunlight and aerobic conditions, it is important to investigate both the chemical and morphological changes of the ligands over time. Changes in the chemical nature and the morphology of the ligand layer have direct implications for its functionality as a separator of the NPs, their adhesion to the substrate, and the interaction with water vapor (through changes of the hydrophilicity of the functional groups) and thus also on device performance under realistic conditions. The direct investigation of the changes induced through oxidation of the NP ligand layer is thus important for a better understanding of their functionality under environmental conditions.

In the present work, we monitor the oxidative degradation of the oleic acid ligand layer surrounding NaYF_4_ nanoparticles (NPs). When doped with lanthanide ions such as Tm^3+^, NaYF_4_ nanoparticles can facilitate photon upconversion^3,4^ and photon avalanching,^5−7^ whose applications include microscale lasing^8^ and subdiffraction imaging.^5,6^ These NPs can also facilitate downconversion as scintillators for X-ray^9^ and electron imaging.^10^ Here, we focus on the oleic acid ligand capping layer of the NPs and its oxidation and partial volatilization by reactive oxidizers. The NaYF_4_ cores act both as a support for the oleic acid ligands and as an X-ray photon absorber and hence as a generator of slow secondary electrons. By considering the total photoionization cross sections^11^ and the mass density of the particles and the SiO_2_/Si substrate, we estimate that the NPs are active per volume in generating electrons than the substrate by a factor of about 2.5 when illuminated by X-ray photons with an energy of 1000 eV. Secondary electrons are known to generate highly reactive oxidation agents, such as OH radicals and O_3_, in the presence of O_2_ and residual water vapor. These species then drive degradation of the oleic acid ligands through oxidation reactions, leading to a shrinkage of the ligand layer through oxidative volatilization. At the same time, the chemical nature of the ligand layer changes due to the formation of new functional groups by the oxygenation of the oleic acid molecules.

Ambient pressure X-ray photoelectron spectroscopy (APXPS) was used in past investigations to monitor the reaction of carbonaceous species (specifically coronene) with highly reactive oxidizing trace gases, such as OH radicals and O_3_.^12^ The chemical and surface sensitivity of XPS allowed us to distinguish different functional groups in C 1s spectra and also to determine the total carbon loss through volatilization of coronene and its oxidation products. One complication in this previous study was that the morphology of the reacted film could not be monitored directly, which introduces uncertainties in the analysis of the APXPS data. In the present work, we address this issue by combining APXPS measurements with grazing incidence X-ray scattering (GIXS) in situ, which provides information about morphological changes to the NPs and their oleic acid ligand layer and is thus complementary to APXPS.^13^

The results of our APXPS and GIXS study show that oxidation of the oleic acid layer leads to the formation of alcohol, carbonyl, and acid groups and that the overall volatilization of oleic acid is the dominating process. GIXS data indicate that the reaction preferentially takes place on the side of the NPs facing the gas phase, ultimately resulting in a chemically and morphologically asymmetric ligand layer, where the oxidized part shows mainly CH_*x*_ and acid group carbons. We believe that the present investigation is a model for in-depth studies of a wide range of reactions that affect both the chemical nature and the morphology of ligand-capped nanomaterials and other carbonaceous nanoscale systems using complementary, surface-sensitive spectroscopic (APXPS) and scattering (GIXS) methods.

## 2 Results and Discussion

The oxidation of the oleic acid layer surrounding the 9 nm NaYF_4_ nanoparticles was monitored using APXPS as a function of O_2_ background pressure and time. For each measurement, a freshly prepared sample was used. The as-prepared samples were first characterized under vacuum by XPS and GIXS. Afterward, O_2_ was admitted to the sample compartment, and the reaction was monitored over time using XPS, with a focus on the C 1s spectra which report on the formation of oxygenated products and the overall volatilization of the carbonaceous layer over time. While an external ozone generator could be used to facilitate the oxidation reaction, it turned out that photodissociation of O_2_ (and residual H_2_O) at submbar pressure, as well as electron-impact excitation and dissociation of O_2_ (and likely residual H_2_O), produced a sufficient amount of reactive oxygenated species for the volatilization of the oleic acid NP layer.

### 2.1 Ambient Pressure X-ray Photoelectron Spectroscopy

**Figure 1:**
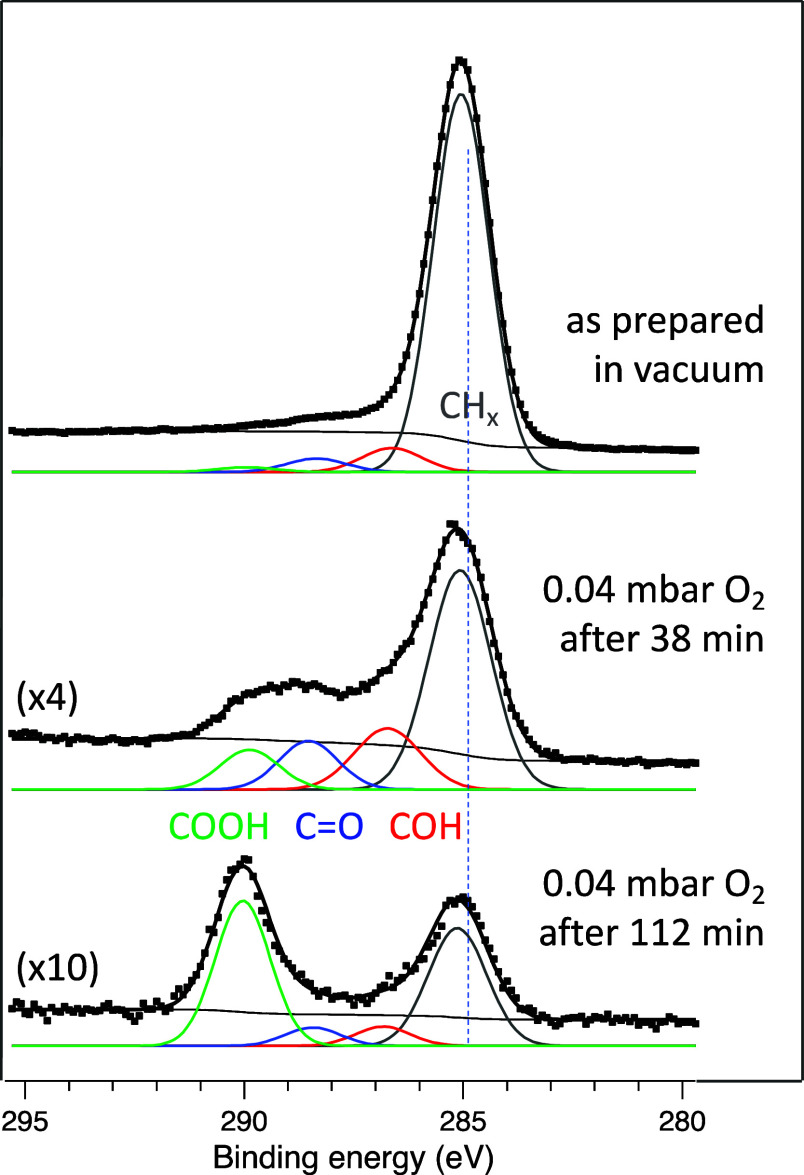
C 1s spectra of NaYF_4_ NPs surrounded by an oleic acid layer and deposited on a SiOx/Si wafer. The top spectrum was taken under vacuum before oxidation. It is dominated by the CH_*x*_ peak of oleic acid, with small traces of oxygenated species already present, which can possibly also arise from adventitious carbon on the substrate. The middle and bottom spectra were recorded during the X-ray beam-assisted oxidation in 0.04 mbar O_2_. The growth of the oxidation products (COH, C=O, and COOH) can be observed alongside the reduction of the CH_*x*_ peak. The BE axis was corrected so that the CH_*x*_ peak of the as-prepared sample is at 285 eV, the literature value. Please note the different intensity scaling of the spectra.

Figure 1 shows the C 1s spectra taken before (top), during (middle), and at the end (bottom) of the oxidation reaction, here for 9 nm NaYF_4_ NPs in 0.04 mbar of O_2_. The spectrum of the as-prepared sample is dominated by the CH_*x*_ peak (at 285 eV), as expected from the chemistry of oleic acid, i.e., C_17_H_33_–COOH. Some amount of oxidized carbon is also present on the sample, which could be due to adventitious carbon and to a smaller part due to the acid groups of oleic acid, which have a BE of ∼290 eV.

**Figure 2:**
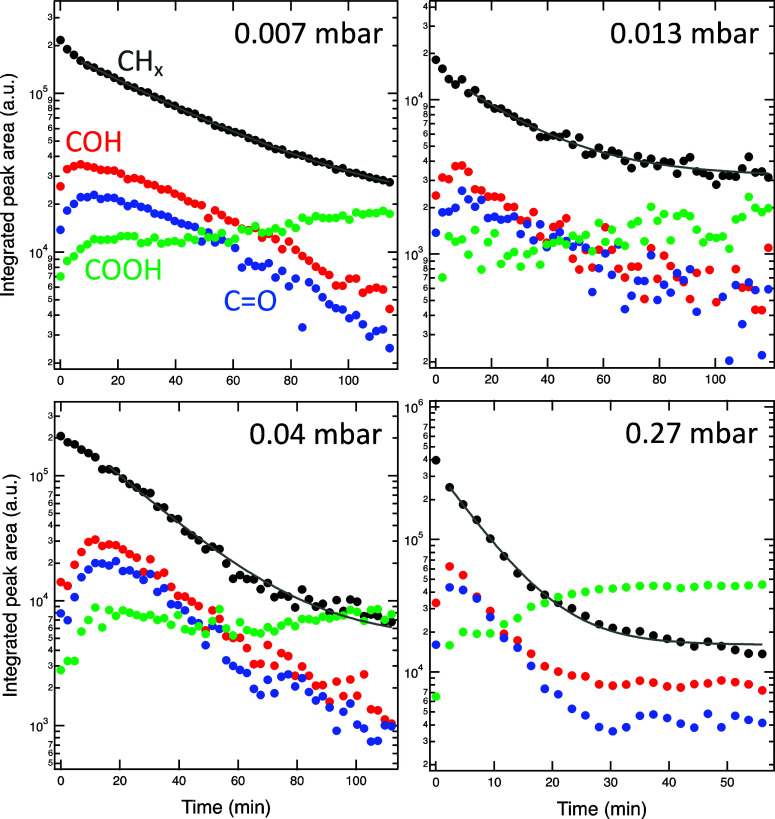
Integrated peak area for the four component peaks in the C 1s spectrum as shown in Figure 1 during oxidation at four different O_2_ pressures. In each case, a continuous decrease of the CH_*x*_ species is observed, while the oxygenated products first grow in intensity and then decrease as well. The solid lines are exponential fits of the decay in the CH_*x*_ signal. Please note that the time and peak area axes do not have the same range for the four different plots.

Once O_2_ is introduced to the chamber, the oxidation of oleic acid (and the adventitious carbon) proceeds readily, as can be seen in the spectra taken after 38 and 112 min in the presence of O_2_. Three product peaks can be clearly distinguished, with binding energies consistent with those for alcohol (∼286.5 eV), carbonyl (∼288 eV), and carboxylic acid (∼290 eV) groups.^14^ The temporal evolution of these species alongside the reduction of the amount of CH_*x*_ is shown in Figure 2 for experiments at four different O_2_ pressures between 0.007 and 0.27 mbar. It is apparent that there is a generational evolution of the different products in the order of COH–C=O–COOH, as previously observed for the case of the oxidation of coronene.^12^ As the oxidation advances, the COOH species become the most abundant species together with some residual CH_*x*_. The ratio of COOH/CH_*x*_ at the end of the oxidation depends on the O_2_ pressure, as does the rate of the oxidation, which increases with O_2_ pressure.

**Figure 3:**
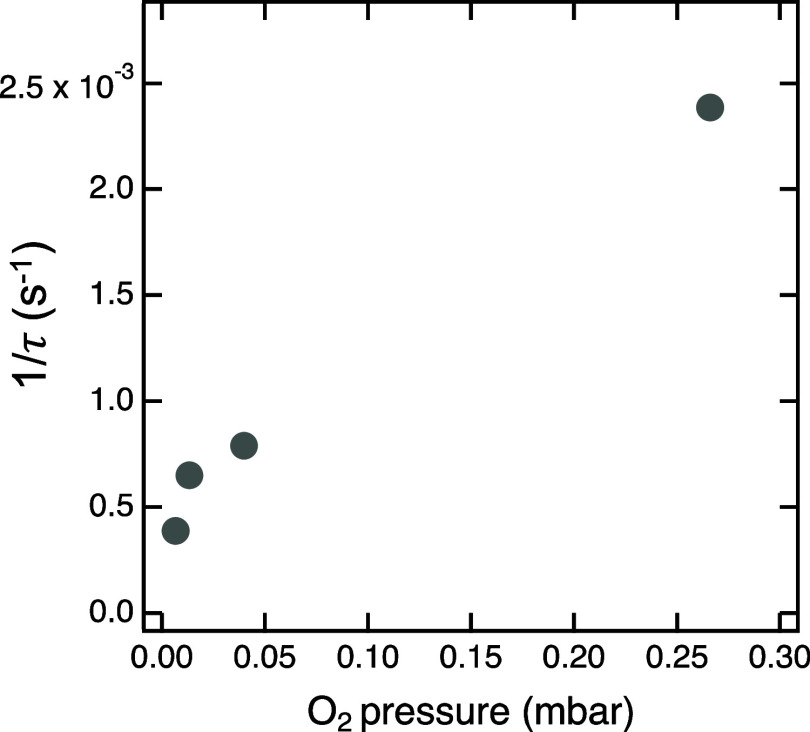
Inverse time constant of the decay of the CH_*x*_ peak as a function of O_2_ background pressure.

To quantify the dependence of the reaction rate on the pressure, we have fitted an exponential dependence to the temporal evolution of the CH_*x*_ signal, shown as solid lines in Figure 2. The inverse time constants determined from these fits are plotted in Figure 3 as a function of the O_2_ pressure. There is a strong indication of a linear dependence between these two quantities, which implies a first-order dependence of the reaction rate on the concentration of reactive oxygen species. This is reasonable considering that the availability of reactive species directly depends on the number of O_2_ molecules in the gas phase, no matter if the process of their creation is electron impact ionization due to secondary, Auger and photoelectrons from the sample and gas, or direct photoionization of the gas phase by the incident X-rays. All other conditions, such as temperature, incident photon flux and energy, and sample composition (which is important for the number and energy distributions of electrons), are similar across these experiments. Plots of the total carbon content as well as the total carbon and oxygen content in the ligand layer as a function of reaction time (see Figure S12 in the Supporting Information) show that volatilization is the dominating process and growth of the ligand layer through functionalization in the early stages of oxidation seem to be negligible, unlike in the case of coronene.^12^ The C/O ratio versus time approaches unity (Figure S13 in the Supporting Information) in all cases, which is in line with the formation of oxidation products with an average stoichiometry akin to that in acetic acid.

### 2.2 Grazing Incidence X-ray Scattering

**Figure 4:**
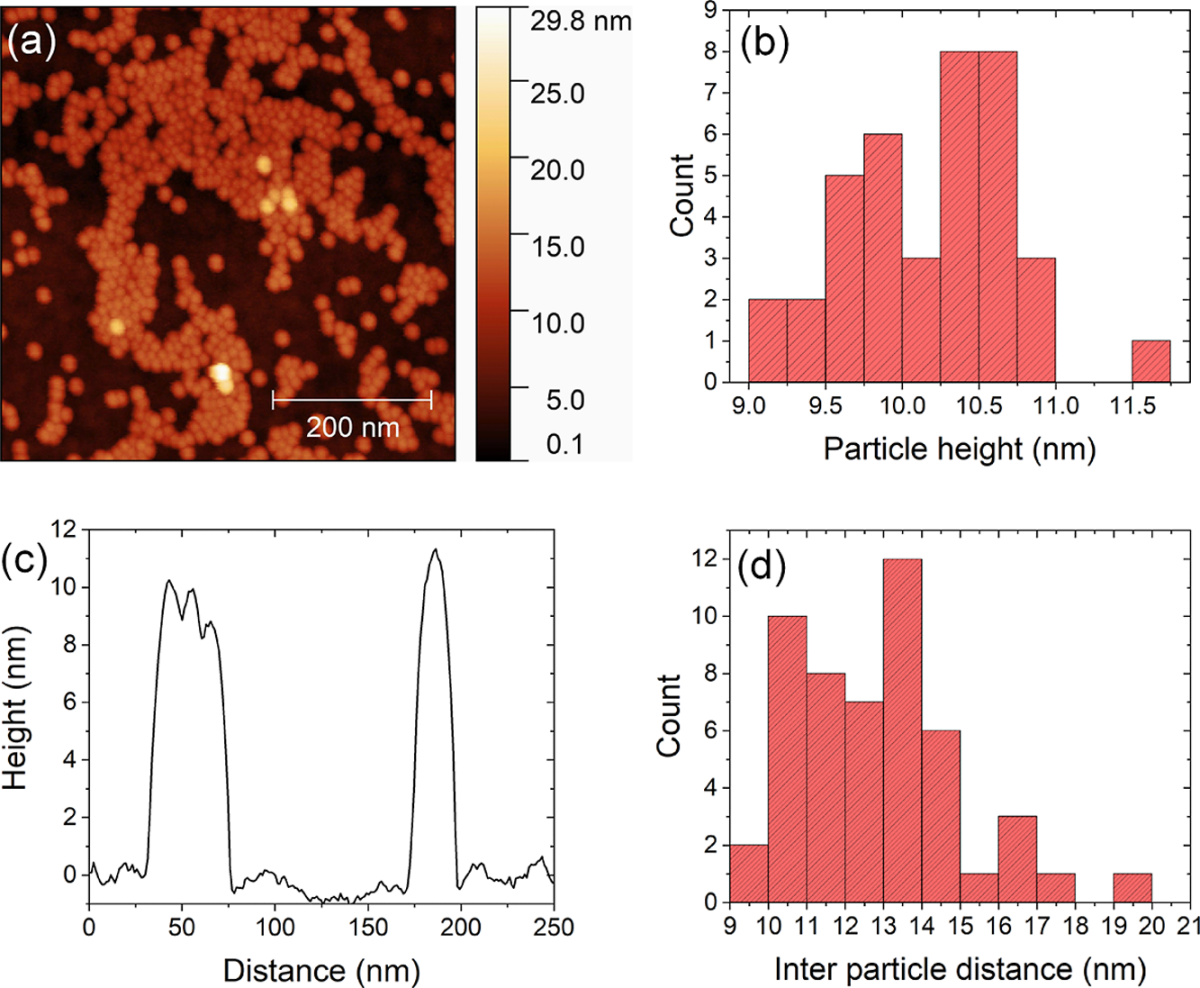
(a) Topography of the as-prepared sample used in the reaction at 0.007 mbar O_2_, characterized by AFM. The NPs are located in two layers, where the bottom layer has a coverage of ∼45%. The second layer (height between 10 and 20 nm) comprises mostly isolated single NPs. (b) The average height of the NPs in the bottom layer, as determined by AFM, is 10.2 ± 0.5 nm. (c) Topographic line scan showing the profile of a single NP (at ∼170 nm) and of clustered NPs (at ∼50 nm), where the NP shape is convoluted with that of the AFM tip. (d) Distribution of the in-plane separation of NPs, showing an average particle spacing of 12.9 ± 2.1 nm.

We now turn our attention to investigating the morphology of the NPs and the oleic acid ligand layer. Ex situ AFM measurements on as-prepared samples (Figure 4) reveal a coverage of approximately one monolayer of the NPs in a closely packed configuration. Occasionally, some NPs are observed in the second layer, confirmed also by the in situ GIXS measurements shown later. The mean particle height and the interparticle distances determined by AFM are 10 and 13 nm, respectively, which is a reasonable value for 9 nm NaYF_4_ NPs covered by oleic acid ligands.

**Figure 5:**
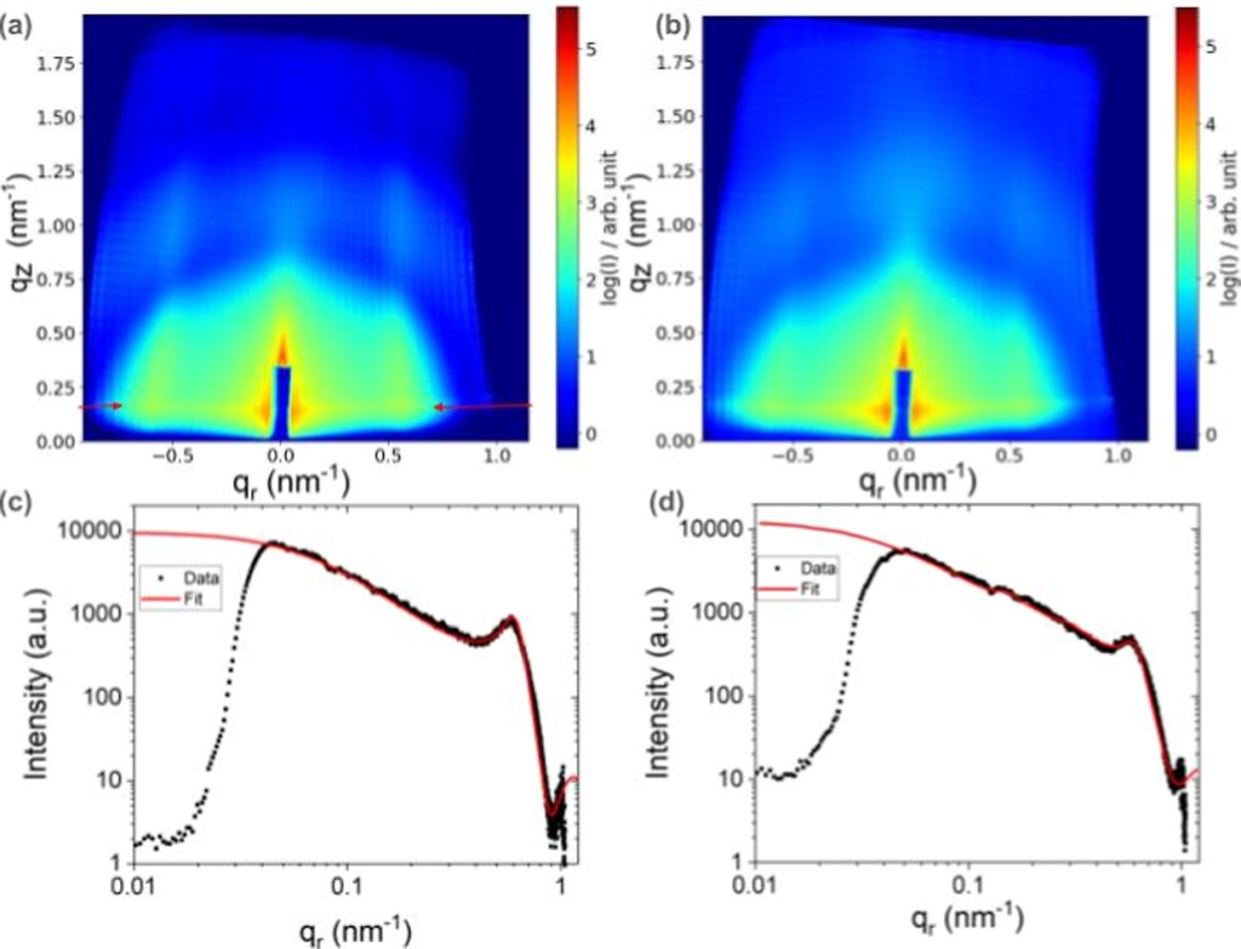
(a,b) X-ray scattering pattern of NaYF_4_/oleic acid ligand NPs measured with an incident photon energy of 1240 eV before and after oxidizing treatment in 0.007 mbar, respectively. The nominal diameter of the NPs is 9.6 nm. (c,d): Analytical fit of a line cut through the scattering data along the Yoneda line (indicated in A by two arrows) of patterns shown in (a,b). The scattering data are modeled assuming closely packed spheres with diameters of 13 and 11 nm, respectively. The q-range below ∼0.5 nm^–1^ is dominated by the NPs’ form factor, while the range above 0.5 nm^–1^ is due to the structure factor. The reduction of the NP size from 13 to 11 nm models the reduction of the oleic acid ligand layer thickness due to the oxidation reaction.

In situ GIXS patterns measured before and after oxidation in the presence of 0.007 mbar O_2_ are shown in Figure 5a,b. The changes in the pattern after oxidation are pronounced and manifest themselves in an overall broadening of the vertical rods, which is mostly due to changes in the structure factor. Changes are also observed for the Yoneda line, which is a horizontal high-intensity line observed at the critical angle, as indicated by arrows in Figure 5a.

An analytical fit of line cuts along the Yoneda line utilizing a core–shell form factor model,^15^ a structure factor for a face-centered cubic (fcc) pattern,^16,17^ and a fractal from factor^18^ reveals a decrease of the overall NP size from 13 to 11 nm upon oxidation (see Figure 5c,d). Please note that the analysis at *q*_r_ values below 4 × 10^–2^ nm^–1^ is not possible due to the presence of the zero order beam stop, and thus data points for these *q* values are not included in the fit. The model used to represent the sample is based on closely packed spherical capped nanoparticles with a nominal diameter of the NaYF_4_ core of 9.6 nm. The decrease in the ligand layer thickness is a direct consequence of the volatilization of oleic acid and its reaction products during the oxidation, as observed by APXPS (see Figures 1 and 2). The overall broadening of the features in the GIXS map of the reacted sample points to an increase in the polydispersity, both in terms of size and shape of the NPs and the spacing between them. More details on the fit methodology are available in Section 1 (analytical model description) of the Supporting Information

**Figure 6:**
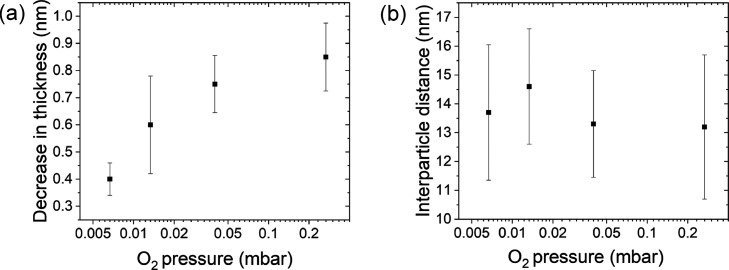
(a) Average reduction in the thickness of the carbonaceous layer around the NaYF_4_ core. The error bars are based on the polydispersity. (b) Interparticle distance (solid squares) and particle displacement (error bars) in the particle fcc lattice during O_2_ treatment as a function of pressure. The data are extracted from in-plane 1D fits of the Yoneda line, as shown for the 0.007 mbar O_2_ measurement in Figure 5D. The equivalent fits for the other O_2_ pressures are shown in the Supporting Information in the section “analytical model description” and in Figures S1 and S2.

We have performed the fit analysis shown in Figure 5 for the case of the measurements at 0.007 mbar and also for the other pressures. The reduction in the thickness of the organic ligand layer and the interparticle distance on the O_2_ pressure are shown in Figure 6a. The data in the left panel imply that the reduction in the organic layer thickness approaches a limiting value at the highest O_2_ pressure of 0.27 mbar. This is in agreement with the APXPS data in Figure 2, which show for the 0.27 mbar oxidation a steady state of the C 1s components already halfway through the experiment after about 30 min.

The analysis of the interparticle distances after the reaction based on GIXS measurements, shown in Figure 6b, indicates that the NPs appear to be firmly bonded to the SiO_*x*_ substrate and still maintain their oleic acid ligand layer in the horizontal plane, since the original in-plane interparticle distance of ∼13.5 nm is still observed after the reaction. This distance is in good agreement with the initial ex situ AFM measurements (Figure 4). This observation also implies that most of the changes to the oleic acid ligand layer are taking place in the out-of-plane direction, as will be discussed next.

**Figure 7:**
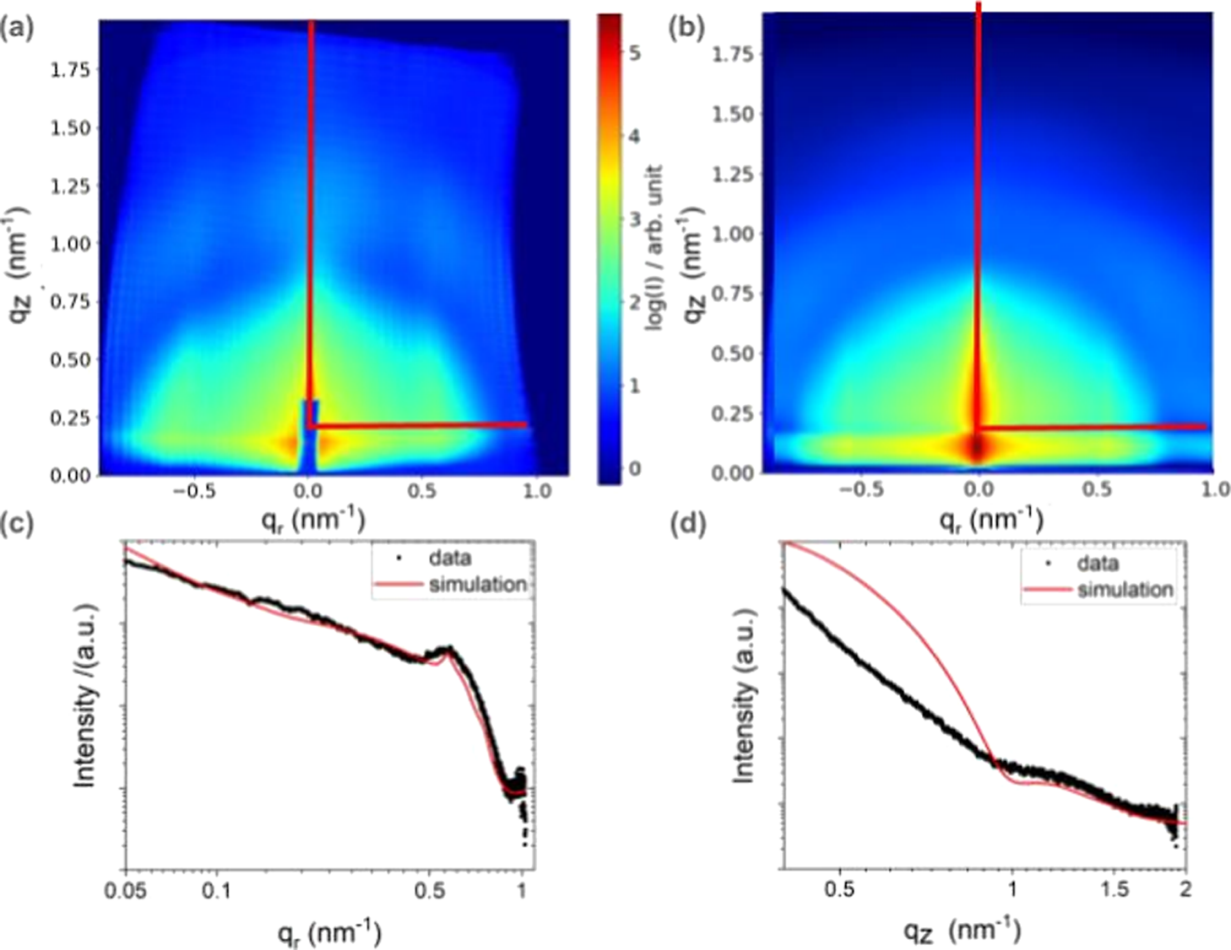
(a) Measured and (b) simulated X-ray scattering pattern of the sample after oxidation in 0.007 mbar O_2_. (c) Horizontal line cuts along the Yoneda line and (d) vertical line cuts along the scattering plane of the experimental and simulated data showing quantitative agreement in the position of the minima and maxima of the line cuts and their slope. The position of the line cuts are indicated in (a,b). The black lines in (c,d) are the experimental data, and the red lines are the results of the simulation.

We now turn our attention to the analysis of the measured X-ray scattering data by comparing them with theoretically predicted scattering patterns based on the simulation of structural models for the NP size, shape, and distribution. Figure 7 shows the analysis on the example of the sample oxidized in 0.007 mbar O_2_. In Figure 7a, the experimental data are displayed, while Figure 7b shows calculated data utilizing the BornAgain simulation package.^19−21^ The simulated GIXS map in Figure S4A in the Supporting Information and in Figure 7b, based on our model (discussed in the next paragraph), shows all the characteristic features of the experimental data, namely, a strong enhancement of the in-plane signal along the Yoneda line, a diffuse ring arising from the 3D form factor of the ligand-covered NPs, and vertical Bragg rods originating from the in-plane structure factor due to the most prevalent population of interparticle distances.

The quantitative comparison of theoretical and experimental data is based on line cuts along the horizontal Yoneda line and the vertical scattering plane. This quantitative comparison is shown in Figure 7c,d. The agreement between the simulated and measured data is very good, especially for larger *q*_r_ and *q*_z_ values, which mainly reflect the properties of the individual NPs and their distances from each other. However, due to restrictions in the input geometry of the state-of-the-art GIXS simulation packages,^21−23^ the reconstructed data can reproduce experimental data only partially, as fcc packing distribution, variations in particle–substrate distance, and particle clustering cannot be simulated alongside particle size distributions.^24,25^ For example, deviation for lower *q*_z_ (out-of-plane direction, Figure 7c) is due to the reflectivity contribution and the roughness of the substrate, which are not included in the numerical simulations. More details on sensitivity of fit between simulated and experimental data on specific model parameters are shown in the Section 2 of the Supporting Information (Figures S4–S8).

The details of the proposed model are discussed next. Initially, the substrate is covered with a 4 nm-thick (possibly noncontinuous) layer of a carbonaceous layer with a mean/effective density of 1/4 of the density of the nominal oleic acid value. This layer is formed by the oleic acid excess, and the NPs closest to the substrate are submerged in this film (Supporting Information Figure S3C). Based on the simulation results, the mass density of the oleic acid in the ligand layer is similar to the density of pure oleic acid,^26^ while the density of the NaYF_4_ nanoparticles was found to be 20% lower than in literature reports.^27^

**Figure 8:**
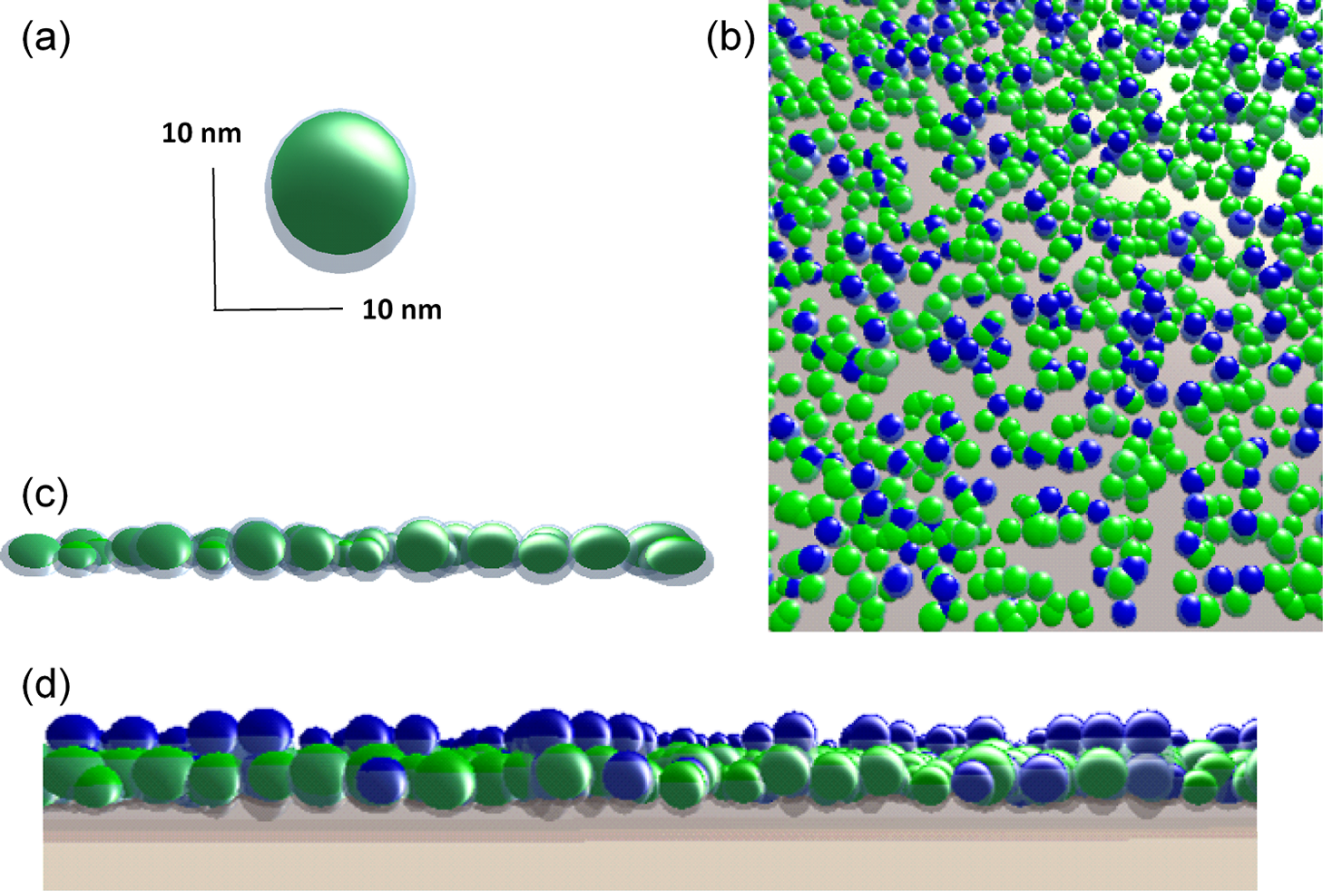
(a) Visualization of a single NP with the capping layer, after oxidation. The asymmetrical shape of the capping layer is a consequence of exposure to O_2_, with limited reaction rates in plane and at the contact to the substrate. (b) 3D model used to simulate the scattering pattern. Particles in the 1st layer are closely packed in a quasi-regular hexagonal pattern with 45% coverage. The second layer is partially filled with quasi-regular in-plane stacking. All NaYF_4_ particles are fully covered by an oleic acid ligand layer with a thickness of 0.6 nm thickness. Due to clustering and other changes of morphology, the NPs have a distribution of diameters, simulated by a Gaussian with ±1 nm fwhm. (c) Graphical depiction of the particle size distribution utilized for the simulation. (d) Side view of the simulation geometry. Particles are lifted off the substrate as they are suspended in the excess capping agent layer. The height of the different fcc stacking is varied between 2.5 nm (blue) and 0.6 nm (green).

Figure 8 shows the model used for the 0.007 mbar O_2_-treated sample after oxidation. The polydispersity and the size of nanoparticles determined from X-ray scattering (9.4 ± 0.6 nm) are in excellent agreement with NP dimensions determined in scanning transmission electron microscopy (not shown) measurements (9.4 ± 0.4 nm). More details on how the individual layers in the Figure 8 model were defined are in the Supporting Information—Section S3.

During oxidation, the thickness of the carbonaceous layer covering the NaYF_4_ core decreases from 1.3 nm (as-prepared oleic acid layer) to 0.8 nm (oxidation products). The majority of the carbonaceous material is removed at the top of the particles, while the ligand layer at the bottom of the NPs (in contact with the substrate) shows little thinning (see Figure 8a). During the oxidation process, the adventitious carbon layer initially covering the substrate is also fully removed according to the results of the comparison of simulated and measured data.

Based on the analysis of the in-plane lattice spacing between NPs (as shown in Figure 8b), there are several populations present both before and after oxidation. In the as-prepared sample, the majority of the NP species exhibits a mean in-plane distance of 12.9 nm and a lattice displacement of 1.25 nm; a minority population of the as-prepared NPs exhibits a broad range of spacing ranging from 10 to 30 nm with large relative displacements (more than 10%), creating a pseudorandomly ordered population. After oxidation, the in-plane lattice spacing of the majority NP population decreases to 12.5 nm, and the particle polydispersity increases from 0.5 to 0.7 (Figure 8c). For both as-prepared and oxidized samples, some NP pairs are observed that share a carbonaceous layer. This is, however, only a small fraction (∼1%) of the total NP population.

From the analysis of the out-of-plane line cuts, it is deduced that there is a relatively large range of substrate–NP distances. This is caused by the formation of a second layer of NPs on top of the monolayer, as well as by the asymmetrical position of some of the topmost NPs within their ligand shell after oxygen exposure. The preferred configuration of the NPs in the second layer is at the bridge site between pairs of particles in the bottom layer. The bottom layer of particles varies in their positions over the substrate between 0 and 6 nm (Figure 8d). This distance variation is substantially reduced after O_2_ exposure to 3 nm due to the volatilization of the excess oleic acid layer during oxidation.

Interestingly, upon completely removing the top layer and setting the particle to substrate distance to 0 nm, the structure factor emerges more prominent, exhibiting a recognizable minimum and broader maximum, but leads to large deviations in the out-of-plane scattering (see Figure S10). Therefore, we suspect that parts of the irradiated nanoparticle film do not exhibit a top layer, as described in Figure 8.

Despite the simplifications used in the model and the existence of other structures that yield similar scattering patterns, the simulated particle sizes, polydispersity, thickness of capping agent layer, average in plane particle distance, and existence of two layers are dependent on highly indicative features like the position and width of the powder ring, the *q* position of the structure factor rods, and the slope around the first minimum of the form factor. Therefore, and due to the good agreement between simulated data and the analytical approach, we are confident that the parameters mentioned above are correctly reflected in the simulation of the scattering data. Additional verification is given by the electron density reconstruction of the out-of-plane line scans that show a significant thinning of the oleic acid shell out of plane after the O_2_ treatment (see Figure S11).

## 3 Conclusions

A model core–shell nanoparticle composed of an inorganic NaYF_4_ core and an oleic acid ligand layer was studied in situ by complementary APXPS and GIXS, revealing the chemical and morphological transformation of the ligand layer due to oxidation. From APXPS data, it was determined that the reaction rate depends linearly on the O_2_ partial pressure, suggesting a first-order transformation of oleic acid into several reaction intermediates, including alcohol, carbonyl, and carboxylic acid groups, the latter ones representing the majority species at the end of the reaction. Most of the ligand film is removed during the oxidation process due to volatilization. GIXS data indicate that during the reaction, the NPs remain firmly attached to the Si substrate, while losing appreciable thickness of the oleic acid layer at the side of the NPs facing the gas atmosphere. It was also found that oxidation leads to a restructuring of the NPs, lowering their mean distance. The effect was observed mainly for out-of-plane distances, while the NPs conserved their in-plane positions. In addition, increased clustering of NPs due to oxidation was observed.

The approach presented in this work is applicable to a wide variety of nanoparticles and other monodisperse systems such as emulsions. The combination of the APXPS and GIXS, which provides information on morphological and chemical changes, holds great promise for future multimodal studies in the fields of environmental science, heterogeneous catalysis, photo- and electrochemistry, and beyond.

## 4 Methods

### 4.1 Nanoparticle Synthesis and Sample Preparation

NaYF_4_: 8% Tm^3+^ upconverting nanoparticles (UCNPs) with a mean diameter size of 9.4 nm were synthesized based on a previously reported method.^28^ For a typical synthesis, YCl_3_ (0.92 mmol, 180 mg) and TmCl_3_ (0.08 mmol, 22 mg) were added into a 50 ml three-neck flask, followed by an addition of 6 mL of oleic acid and 14 mL of octadecene. The solution was stirred under vacuum and heated to 100 °C for 1 h. During this time, the solution became clear. After that, the flask was then subjected to three pump–purge cycles, each consisting of refilling with N_2_ and immediately pumping under vacuum to remove water and oxygen. Afterward, sodium oleate (2.5 mmol, 762 mg) and NH_4_F (4 mmol, 148 mg) were added to the flask under a N_2_ flow. Subsequently, the resealed flask was resealed and placed under vacuum for 15 min at 100 °C, followed by three pump–purge cycles. At the end of the treatment, the flask was quickly heated from 100 to 320 °C (the approximate ramp rate was 25 °C min^–1^). The temperature was held at 320 °C for 40 min, after which the flask was rapidly cooled to room temperature by using a stream of compressed air.

To isolate the nanoparticles, ethanol was added to the solution in a 1:1 volume ratio, and the precipitated nanoparticles were isolated by centrifugation (5 min at 4000 rpm). The pellet was suspended in hexanes and centrifuged to remove large, aggregated particles. The nanoparticles remaining in the supernatant were washed two additional times by adding ethanol, isolating by centrifugation, and dissolving the pellet in hexanes. The nanoparticles were stored in hexanes.

The NPs were deposited onto Si wafers by immersing a Si wafer piece with dimensions of ∼5 × 20 × 0.2 mm face down into the solution, which was kept in a 5 mL screw cap bottle lying on its side. In this manner, the Si surface did not touch the walls of the tube (only at the outer edges) and the NPs assembled from solution onto the Si surface through adhesive interactions and not gravity. The Si wafers were left immersed in the NP/hexane solutions for a minimum of 10 min before being removed, dried in air, and mounted inside the APXPS instrument without further treatment.

### 4.2 APXPS and GIXS Measurements

The APXPS and GIXS measurements were performed using the APPEXS (Ambient Pressure PhotoElectron spectroscopy and X-ray Scattering) setup at the APXPS-2 port of beamline 11.0.2 of the Advanced Light Source at Lawrence Berkeley National Laboratory in Berkeley, CA.^13^ The incident photon energy was 1000 eV for the APXPS and 1240 eV for the GIXS maps, with an exit slit size of 60 × 250 μm. The resulting photon flux at these energies is estimated to be 3 × 10^11^ photons/sec and 6 × 10^10^ photons/sec, respectively. The combined beamline and electron analyzer resolution in the experiments was better than 0.8 eV. The X-rays were incident on the sample under a grazing angle of ∼2 deg in the proximity of the critical angle of the Si substrate, with the electron detection direction at 15 deg from the sample normal. X-ray scattering data were collected using a 2D CCD detector (Andor iKon-L) mounted approximately 570 mm from the sample surface on a two-axis camera rotating manipulator covering a solid angle of ±12° in plane and +24° out of plane (above the horizon). This corresponds to covering a scattering vector *q*-range of ±1.5 and 3 nm^–1^, respectively. The composite scattering image was reconstructed from 1476 individual images with a *q*-resolution better than 1.5 × 10^–4^ nm^–1^ based on the camera pixel pitch, the camera–sample distance, and X-ray wavelength, excluding effects such as Scherrer domain/grain size.^29^ The total acquisition time of one scattering image was around 80 min, with ca. ∼ 12 min of total exposure (the remaining time is the overhead required for camera movement and readout). We do not observe any significant change of the ligand moiety during the mapping procedure, as XPS data showed that they were collected both before and after the X-ray scattering experiments. The X-ray detector was separated from the reaction chamber by a large-area ultrathin (150 nm) Si_3_N_4_ window matching the area of the detector (27 × 27 mm^2^), assuring that the pressure near the CCD chip is kept below 10^–6^ Torr and the X-ray transmission is sufficiently high even in a soft X-ray regime.

The emphasis of the APXPS measurements was on the C 1s, Y 3p, O 1s, and Si 2p core levels. C 1s and Y 3p (which have very similar binding energies) report on the chemical state of the NP shell and core, respectively, while Si 2p reveals changes to the coverage of the Si substrate by the NPs and possible oxidation of the Si wafer. The O 1s signal stems from both the thin oxide layer on the substrate, the reaction products of the oxidation of oleic acid, and any residual carbon contamination on the Si wafer. The XPS data were deconvoluted by using a commercial software package (KolXPD). Pure Gaussians were sufficient to fit all of the peaks.

## Data Availability

All relevant data and analysis scripts used in this study are available from the corresponding authors upon reasonable request.

## Supporting Information Available

Details on the analysis of the GIXS data additional results on the time-dependent evolution of the C 1s signal in XPS (PDF)

## Notes

The manuscript was previously published on arXiv: Maximilian Jaugstetter, Xiao Qi, Emory Chan, Miquel Salmeron, Kevin R. Wilson, Slavomír Nemšák, Hendrik Bluhm. Direct Observation of Morphological and Chemical Changes During the Oxidation of Model Inorganic Ligand-Capped Particles. 2024, article number 2407.00598. arXiv physics. https://arxiv.org/abs/2407.00598 (accessed Nov 15, 2024).
