# Supplementary material for: Direct Observation of Morphological and Chemical Changes during the Oxidation of Model Inorganic Ligand-Capped Particles
Source: ACS Nano. 2024 Dec 19;19(1):418–26. doi: 10.1021/acsnano.4c08846 (PMC11752503; doi:10.1021/acsnano.4c08846)
Supplement: Supplementary file 1 — nn4c08846_si_001.pdf [file nn4c08846_si_001.pdf]

Direct Observation of Morphological and Chemical Changes During the Oxidation of Model Inorganic Ligand-Capped Particles

Supplementary Information

Maximilian Jaugstetter[1,*], Xiao Qi[2], Emory M. Chan[2], Miquel Salmeron[1], Kevin R. Wilson[3], Slavomír Nemšák[4,5,*], Hendrik Bluhm[6,*]

[1]*Materials Sciences Division, Lawrence Berkeley National Laboratory, Berkeley, CA 94720, USA*

[2]*Molecular Foundry, Lawrence Berkeley National Laboratory, Berkeley, CA 94720, USA*

[3]*Chemical Sciences Division, Lawrence Berkeley National Laboratory, Berkeley, CA 94720, USA*

[4]*Advanced Light Source, Lawrence Berkeley National Laboratory, Berkeley, CA 94720, USA*

[5]*Department of Physics and Astronomy, University of California, Davis, CA 95616, USA*

[6]*Fritz Haber Institute of the Max Planck Society, D-14195 Berlin, Germany*

***Section 1 – GIXS analytical model description*.**

In-plane cuts were performed at q_z = 0.2 nm^{-1}, which corresponds to the critical angle of 2° of the $NaYF_4$ nanoparticles to maximize the contribution of the nanoparticle to the scattering curve. The critical angle of $NaYF_4$ was calculated using Henke tables[1] at a photon energy of 1240 eV using a chemical formula of $NaYF_4$ and density of 5.578 gcm^{-3} as calculated from the molecular weight data given by Mackenzie et al.[2]

The model used to fit in-plane line cuts (along Yoneda line) consists of a core/shell sphere form factor.[3] Here, the $NaYF_4$ nanoparticles function as the sphere's core and the oleic acid ligand layer as spheres shell. Size distribution is added via a Gaussian distribution model with set average and standard deviation defined by the PD value. A fractal structure factor[4] consisting of the spherical from factor of the solid nanoparticles as single unit and a total equivalent radius of 24 nm with a dimensionality of 2.2 to account for formed pseudo-crystalline assemblies of the deposited nanoparticles. Lastly, 3 FCC structure factors with different spacing[5,6] were used to account for the different populations in the formed

nanoparticle islands (as shown in figure 4 in the main text) on the substrate. Different spacing is given for top layer with a weight of around 10 %, for the interference between particles at the edges of the formed island with another 10 % contribution.

The main structure factor can be observed as shoulders in all measured scattering patterns and appear as peaks in the individual line cuts and is defined by the inter-particle distance within the individual islands. This structure factor contributes 80 % to the total structure factor and shows slightly bigger inter-particle distance than particle size with varying spacing between the samples (see Figure 6 in the main text). Individual line-cuts together with fits are shown in the main text in Figure 5 and Figures S1, S2 and S3.

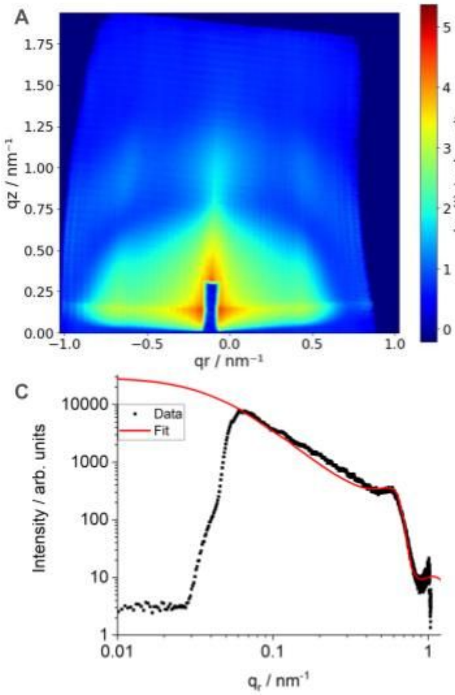

Figure S1: Scattering pattern (**A, B**) and fitted in-plane line (**C, D**) cuts for $NaYF_4$ particles capped with oleic acid before (left) and during treatment with 0.013 mBar O_2 (right).

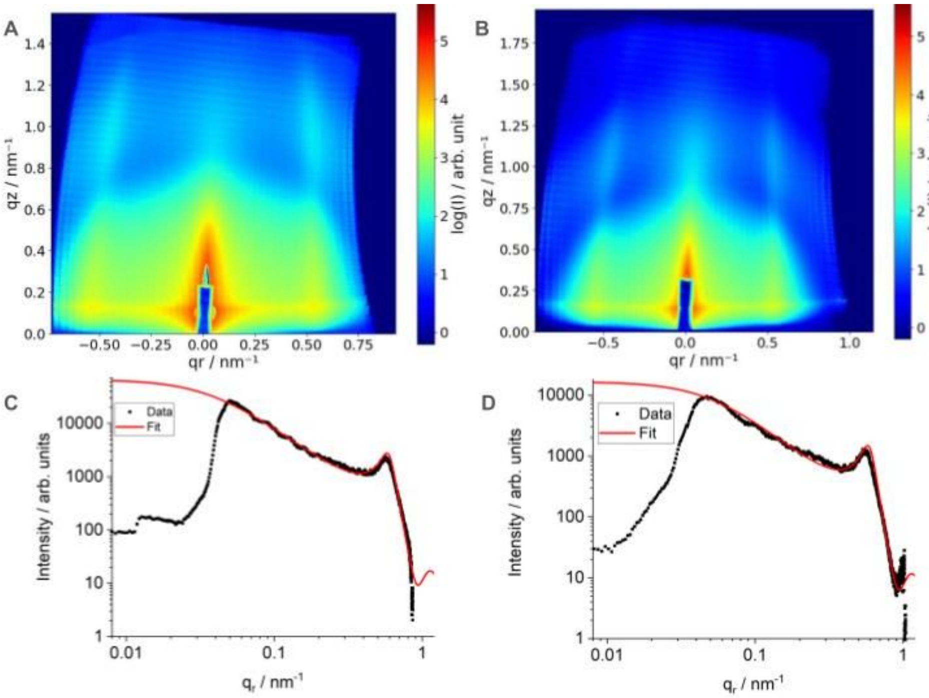

Figure S2: Scattering pattern (A, B) and fitted in-plane line (C, D) cuts for $NaYF_4$ particles capped with oleic acid before (left) and during treatment with 0.04 mBar O_2 (right).

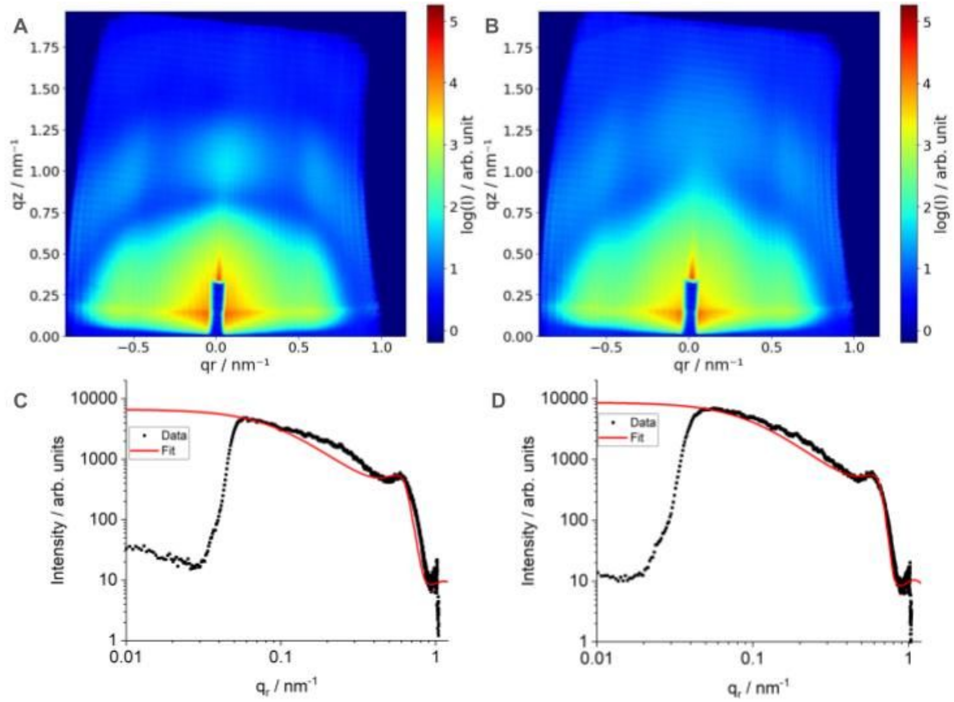

Figure S3: Scattering pattern (**A, B**) and fitted in-plane line (**C, D**) cuts for $NaYF_4$ particles capped with oleic acid before (left) and during treatment with 0.13 mBar O_2 (right).

For a better comparability of the effects of oxygen treatment on the oleic acid moieties, structure factors have been held constant for before and after samples. The core form factor was restricted to 5 % size changes and no scattering length density changes, while the shell fitted without restrictions. The deviations from fitted curves and the data for all samples at q-values around 0.2 nm^{-1} can be explained by the presence of a variety of more broadened structure factors that are caused by the island-like layer growth of the adsorbed particles and not individually fitted. In Figure S2, the sample before oxygen treatment was measured at a photon energy of 1000 eV instead of the 1240 eV for the other samples. This leads to a q vector that is 1.24 times smaller. The evident comparability of the before and after measurements of these samples under two photon energies shows that the used models and parameters can be transferred to modified conditions.

The ability to track changes of the thickness of the oleic acid moiety for the different samples indicates the robustness of the evaluation method used and the preference for the particles form factor at the chosen cut angle. It also speaks for a nonlocal minimum for the used models, as they converge for all samples. This is despite their clear variation in scattering pattern, due to difference in nanoparticle stacking and island size during as a result of variations in coverage.

Section 2 – Simulated X-ray scattering patterns**, assessment of sensitivity**

The optimal fit between simulated and experimental data yields a core radius of 4.9 nm, a core density of 0.55x the reported density of $NaYF_4$ upconversion nanoparticles, a shell thickness of 1.5 nm and a shell density of 1 x oleic acid. Optical constants for the simulated model were extracted from CXRO Henke tables[1] using the data for Si, SiO_2, $NaYF_4$, and oleic acid ($C_{18}H_{34}O_2$, density =0.895 gcm^{-3})[7] at a photon energy of 1240 eV. The simulated particles are placed in islands with a hexagonal closely packed (FCC) lattice structure and a lattice constant of 12 nm (see Figure S4 B). This leads to the appearance of strong secondary peaks at q = 0.52 nm^{-1}, similar to the experimental data (Figure S4 A). The used model includes an adsorbed layer of oleic acid on the substrate material with a thickness of 4 nm. The adsorbed particles are partly sitting on top of this layer or they are suspended in this layer. This leads to two populations of nanoparticle layers with the higher population being 4.1 nm above the substrate and the lower population being about 1.2 nm above the substrate and polydispersity of both core and ligand shell of 0.12 (see Figure S4C). As the particle densities deviated from the literature data, this model was not utilized to fit the shape and structure of the O_2 treated sample as shown in Figure 6 in the main text.

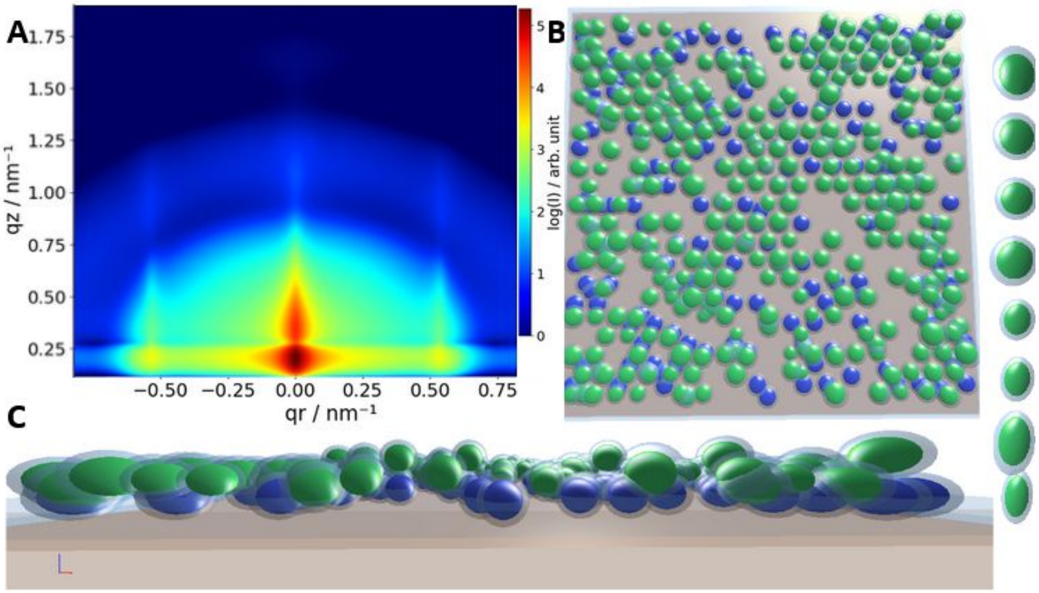

Figure S4: Simulated X-ray scattering pattern (**A**) based on the model shown in (**B**) and (**C**).

To circumvent limitations of the definable geometry in BornAgain simulation package, a workaround with three different interparticle distances of 9.8 nm, 11.6 nm and 12.9 nm and substrate-NP heights of 1.5 nm, 0.8 nm and 0.6 nm in the densely packed first layer was used. The partial coverages of the three layers were 10, 10 and 60 %, respectively. For example, the structure factor at higher out-of-plane scattering vectors (vertical rods) is represented more strongly in the experimental data than in the

simulation. To minimize this discrepancy, particle agglomerates as observed in the AFM images can be added to the model as paracrystals (as used earlier in the analytical fit).

Figure S5A represents the horizontal and vertical line cuts taken from the simulation shown in Figure S4. Figures S5B and S6-S8 demonstrate the sensitivity of different parameter variations on resulting fit. Deviations of the simulated and measured data at high *q* values can be explained by a background radiation coming from Compton scattering that was not added to the simulation to maintain good contrast for the higher order minima in simulated scattering pattern. The deviation at *q* lower than the first order minimum in the out-of-plane scan is not yet fully understood but might be rooted in the geometry of the used scattering setup, as they are apparent in other datasets collected at the APPEXS endstation at beamline 11.0.2 at the ALS.

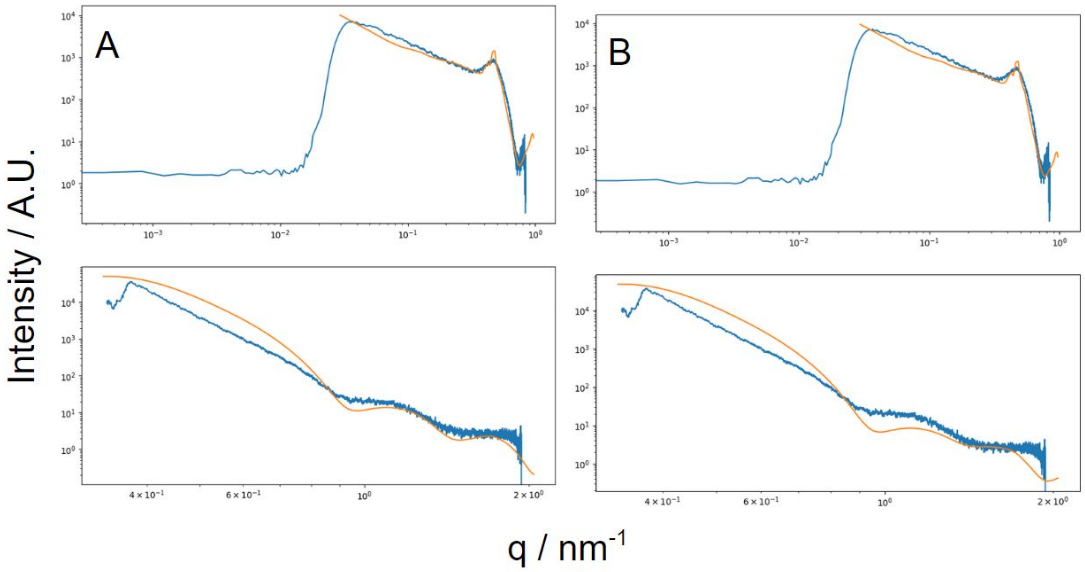

Figure S5: In-plane (top) and out-of-plane line cuts (bottom) taken from the measured scattering pattern before O_2 treatment (blue) and from simulated data (orange). **A** Core diameter of 4.9 nm, a core density of 0.55x the reported density of NaYF4 upconversion nanoparticles, a shell thickness of 1.5 nm and a shell density of 1x oleic acid. **B** Core radius is reduced by 0.4 nm to a total core radius of 4.5 nm.

As shown in Figure S5 B upon decreasing the size of the $NaYF_4$ nanoparticle by 0.4 nm, while maintaining an overall radius of 6.4 nm leads to significant changes in both in-plane and out-of-plane scattering curves. The in-plane curve (Figure S5 B top) shows a shift of the form factor related minimum intensity to higher *q* values and a slight decrease in overall scattering intensity. The out-of-plane curve shows more severe changes as period of the minima is modulated to higher *q* with changing overall particle density and increased thickness of the oleic acid ligand shell.

Upon increasing the ligand layer thickness from 1.3 to 1.7 nm, the overall scattering intensity is slightly reduced, while the first order minimum is shifted to lower *q* values (compare Figure S6 A and B). Additionally, the period between the first and second order minimum becomes longer and the amplitude of the second order minimum increases above experimental values.

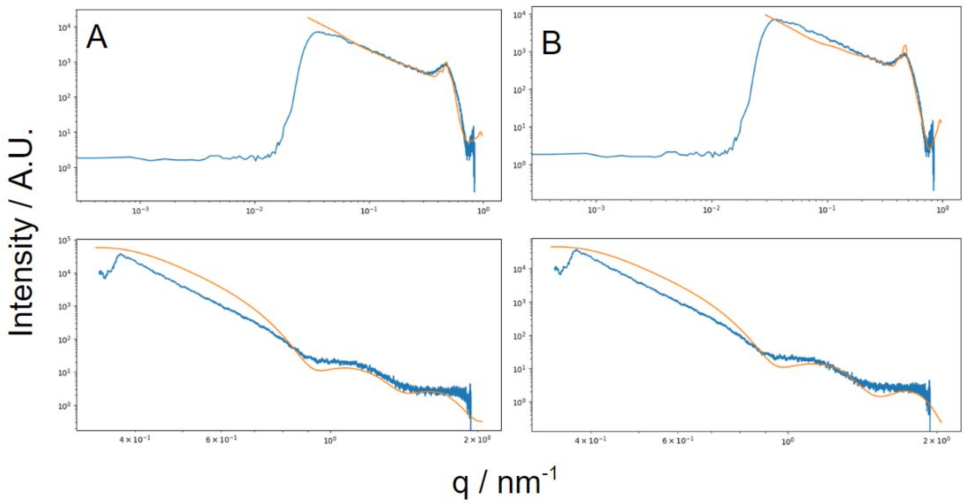

Figure S6: In-plane (top) and out-of-plane line cuts (bottom) taken from the measured scattering pattern before O_2 treatment (blue) and from simulated data (orange). **A** Shell thickness is increased by 0.2 nm to a total core+shell radius of 6.6 nm. **B** Shell thickness is reduced by 0.2 nm to a total core+shell radius of 6.2 nm.

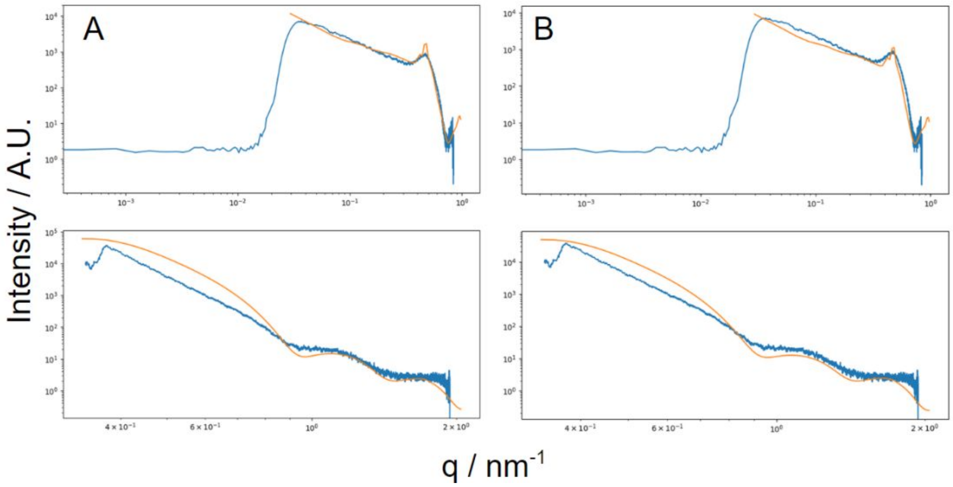

Figure S7: Horizontal (top) and vertical line cuts (bottom) taken from the measured scattering pattern before O_2 treatment (blue) and from simulated data (orange). **A** Core density is increased by 0.05 to a total density of 0.6 x NaYF4. **B** Core density is reduced by 0.05 to a total density of 0.5 x NaYF4

To follow the dependence of the simulated scattering pattern on the density of the nanoparticle and oleic acid moiety, the optical constants were calculated using the procedure described above, while the density was varied. A variation of the optical constants of the $NaYF_4$ nanoparticles leads to an increase/decrease in the total scattering intensity for increased/decreased values (compare Figure S7 A and B). Additionally, the influence of the ligand moiety on the overall form factor increases with decreasing $NaYF_4$ density due to a decreased contrast between nanoparticle and shell, leading to a shift of the first order maximum to higher *q* in both in and out-of-plane scans and vice versa for increased density. Similar effects can be observed with a change in the shell density, as the first order minimum shifts to higher *q*. Additionally, the amplitude of the first order minimum increases due to an increased contrast between particle and ligand shell, while the amplitude of the second order minimum, associated with the overall particle size decreases, as the contrast between vacuum and the ligand decreases (see Figure S8 A). A reduction of the polydispersity for the core/shell form factor leads to more pronounced minima (see Figure S8 B).

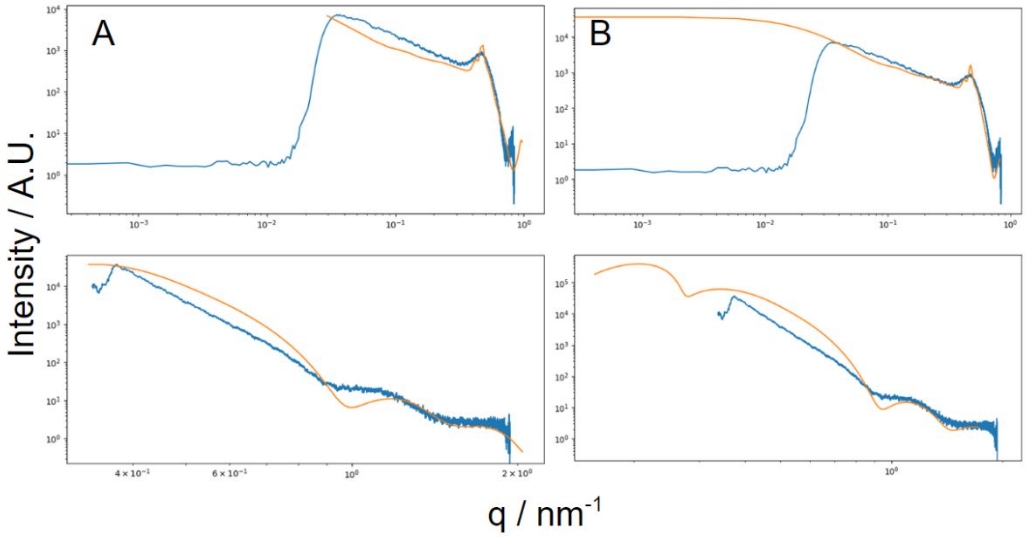

Figure S8: Horizontal (top) and vertical line cuts (bottom) taken from the measured scattering pattern before O_2 treatment (blue) and from simulated data (orange). **A** Shell density is reduced by 0.5 to a total density of 0.5x $NaYF_4$. **B** Core/shell polydispersity is reduced from 0.28 to 0.25

. By comparing the variations of the particle input parameter with their visible influence on the scattering curve we can conclude, within the constraints of the used model, an error of the evaluated particle radius, shell thickness, particle and shell thickness and polydispersity below the demonstrated values. In values, the evaluated size is 4.9 nm ± 0.1 nm, the evaluated thickness 1.6 ± 0.1 nm, the evaluated particle density 3.07 ± 0.14 gcm^{-3}, the shell density 0.895 ± 0.224 gcm^{-3} and the polydispersity 0.28 ± 0.015.

Section 3 – Simulated X-ray scattering pattern after O_2 exposure

To circumvent limitations of the definable geometry in BornAgain simulation package, a workaround with three different interparticle distances of 9.8 nm, 11.6 nm and 12.9 nm and substrate-NP heights of 1.5 nm, 0.8 nm and 0.6 nm in the densely packed first layer was used. The partial coverages of the three layers were 10, 10 and 60 %, respectively. For example, the structure factor at higher out-of-plane scattering vectors (vertical rods) is represented more strongly in the experimental data than in the simulation. To minimize this discrepancy, particle agglomerates as observed in the AFM images can be added to the model as paracrystals (as used earlier in the Section 1 - analytical fitting).

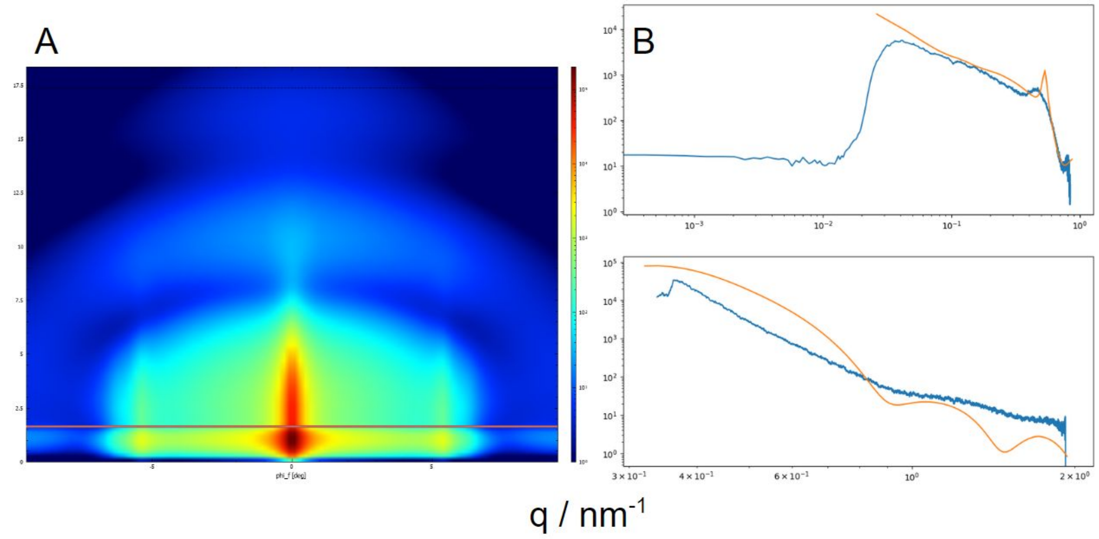

Figure S9: Simulation of oleic acid nanoparticles after O_2 treatment, with focus on maintaining the strong secondary peaks at 5.5 degrees associated with the structure factor. A Simulated scattering pattern. B Comparison between simulated (orange) and measured (blue) line cuts along the horizontal axis (top) and the vertical axis (bottom).

Figure S9 shows a simulated scattering pattern for the oleic acid capped $NaYF_4$ nanoparticle system after a treatment with 0.007 mBar O_2 derived from the same model as shown in Figure S4. The simulation was modified by reducing shell thickness to 0.8 nm, increasing shell density 1.4 times, removing the oleic acid layer on the substrate and decreasing the number of nanoparticles. The big central feature is given by patches of oleic acid on the substrate with a height of 5 nm and an average radius of 15 nm, with a polydispersity of 0.5. The scattering pattern of this model represents the structure factor derived peaks at q=0.52 nm^{-1} visually better than the model in the main text (see Figure S9A). Additionally, the appearance of the second order maximum exhibits more similarities to the recorded data. Despite this, the simulated data does not agree quantitatively both in out-of-plane and in-plane cuts, with the highest deviations in the amplitude of the first and second order minima, and the shape and amplitude of the structure factor maximum.

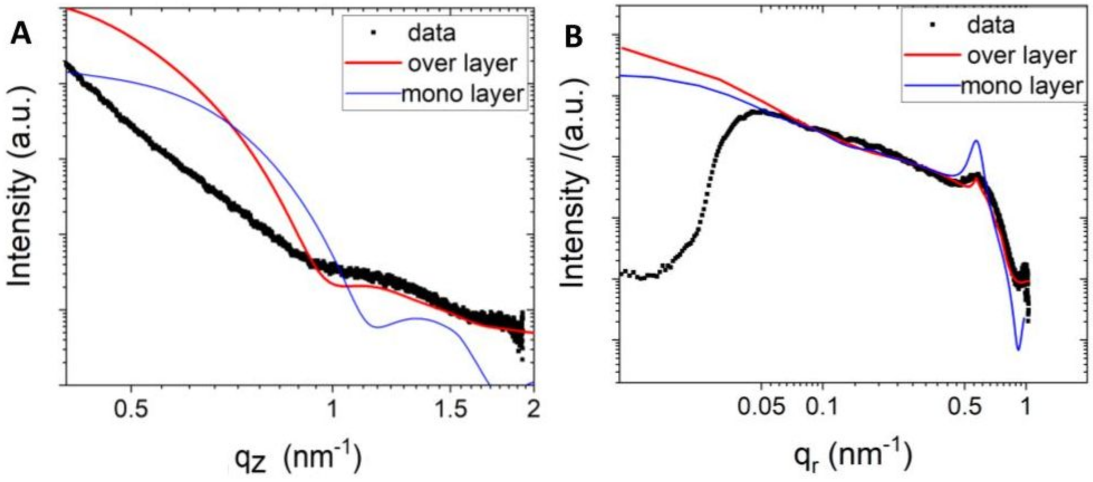

Figure S10: Comparison between the simulated geometry in Figure 8 with (red) and without (blue) a specified second layer and distance to the substrate and the measured data (black). **A** Vertical line cut. **B** Horizontal line cut.

A comparison between the mono layer sample and the sample with an island like top layer as utilized in the main text Figure 7 and 8 shows that the out of plane scattering is shifted to higher q indicating apparently bigger nanoparticles not fitting the experimental data at all (see Figure S10 A). Contrary the horizontal line cut exhibits a wider structure factor and fits the data better in the low q region (see Figure S10 B). Under assumption of different spacing in different irradiated regions this structure factor can be smoothened out to closely resemble the measured data (not shown here). When comparing the two models with the data it is apparent the real geometry contains regions that exhibit a overlayer and a distributed particle substrate distance and regions that can be defined by an FCC monolayer with vacancies. As both geometries cannot be simulated simultaneously and the main focus of this publication lies on the change of the oleic acid capping layer, we focused on the the former as it represents a better average of the sample.

Section 4 – Electron density reconstruction of oleic acid capped NaYF4 nanoparticles

To verify the assumption of the simulated model in figure 7 of the main text, we conducted an independent analysis using an electron density reconstruction of the out-of-plane scattering curve of the O_2 treated sample, shown in figure S5A. To generate the reconstruction, pair distance distribution function (PDDF) of the curve was performed using BioXTAS RAW 2.[8] The used PDDF assumed a monodisperse arbitrary shape, a maximum particle size of 11.4 nm, and the first 200 points were truncated for the fit. A quality of fit value of 71 was reached for the PDDF. Electron density reconstruction was performed using the DENSS code and a total of 200 electrons.[9] The reconstruction yielded a radius of gyration of 4.24 nm and a mean RSC of 0.99 with a low ambiguity of the reconstructed model. The electron density reconstruction was drawn in a volume representation with PYMOL[10] as shown in Figure S10.

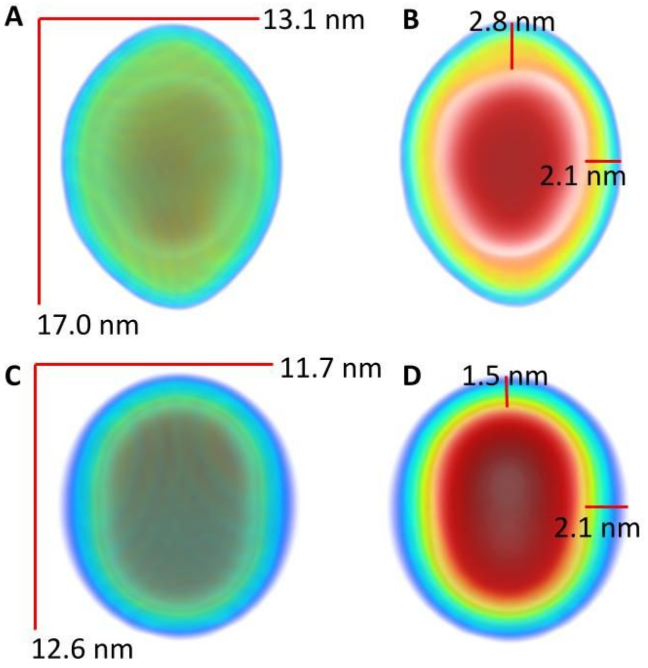

Figure S11: Electron density reconstruction of the out-of-plane scan of 9 nm $NaYF_4$ nanoparticles capped with 1.6 nm oleic acid before and after treatment in 5 mTorr O_2. The color map marks electron density with dark red being more than 12 electrons/voxel, light red between 7 and 12 electrons/voxel, yellow between 7 and 4 electrons/voxel, turquoise between 4 and 1 electrons/voxel, and dark blue below 1 electron/voxel. A,C Volume representation with scale bar derived from the radius of gyration of the particle before and after O_2 treatment, respectively. B,D Slice representation of the particle center with indicators for the thickness of the oleic acid layer, before and after O_2 treatment, respectively.

The electron density reconstruction reveals an elongated nanoparticle with a lower shell thickness on the equatorial axes when compared to the longitudinal axes (see Figure S11 B) before O_2 treatment and a reversed behavior afterwards (see Figure S11 D). This structure confirms the two main assumptions of the simulation model shown in the main text.

First, with the knowledge that the NaYF4 nanoparticles are nearly perfectly spherical, derived from AFM, TEM and SEM the elongated shape implied different distances of the particle center from the substrate as the interaction between reflectivity and form factor decreases the q value of the first order minimum for particles sitting further away from the surface, leading to an apparent increase in particle size in the out-of-plane direction. This effect is more apparent in Figure S11 A when compared to Figure S11 C as the top particle layer is lifted further away from the substrate, further validating the assumptions of the BornAgain simulation.

Second, the shell in the elongated direction is expected to be thicker due to the electron density smearing originating from the particle displacement. This is the case for the reconstruction of the particles before O_2 treatment. After the treatment the shell along elongated axis is thinner, this implies a significant higher in-plane thickness and therefore an inhomogeneous etching of the ligand moiety under O_2 influence, with the highest rates directly on top of the particles. As the DENSS reconstruction is an ab initio method and not fed with any assumption from the simulation, this similarity in results implies that the used model and drawn conclusions reflect the correct geometry with a robust accuracy.

Section 5 – Additional information from C 1s APXPS spectra

In the main manuscript, the evolution of the functional groups (CH_x, COH, C=O, COOH) as a function of oxidation time is shown for the four different O_2 pressures (Figure 2). For completeness, we show in the following additional information that can be extracted from C 1s spectra (such as those shown in Figure 1 of the main manuscript).

Figure S12a displays the total carbon amount for the four different experiments as a function of reaction time. This value was calculated by adding up the four C 1s components (CH_x, COH, C=O, COOH). The data of each pressure series are normalized to unity for a data point taken at the beginning of the oxidation reaction, after about 4 min. Fig. S9a shows a decrease in the amount of carbon with reaction time, in agreement with a gradual volatilization of the ligand layer around the NPs.

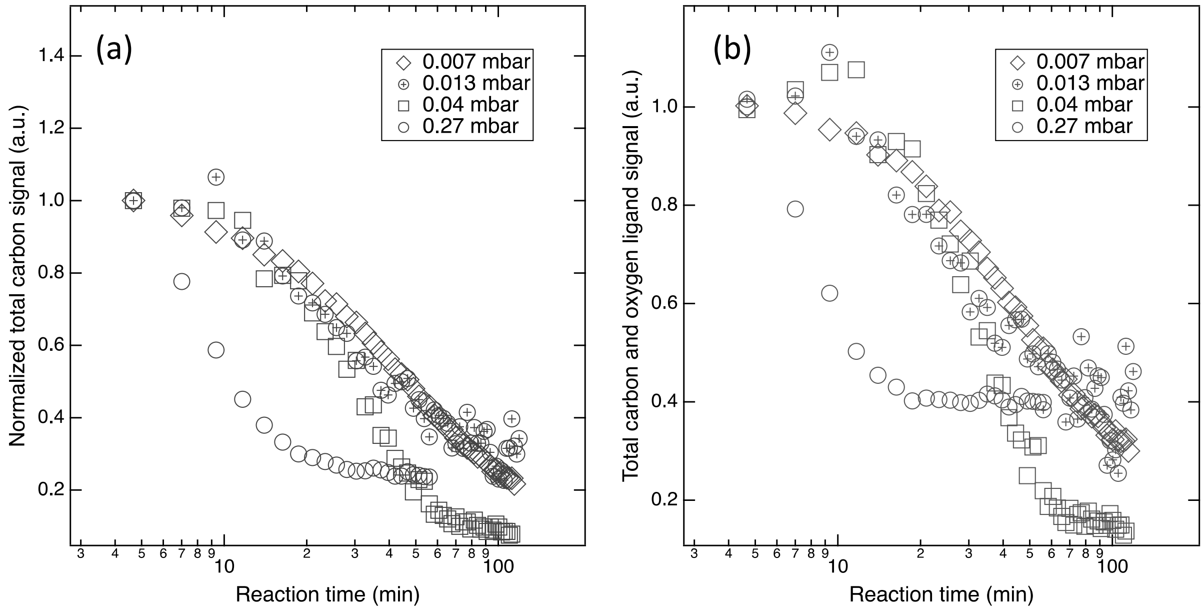

Figure S12. (a) Total C 1s signal as a function of reaction time for the four different O_2 reaction pressures. **(b)** Total carbon and oxygen content deduced from the C 1s signal, under consideration of the C/O stoichiometry of the different C species.

The data in Fig. S12a are not sufficient to judge the change in the thickness of the ligand layer with reaction time. For this, the addition of oxygen due to the functionalization in the oxidation process needs to be taken into account. The total amount of material in the ligand layer (neglecting hydrogens) is then calculated from the C 1s peak components areas according to $(1*CH_x)+(2*COH)+(2*C=O)+(3*COOH)$, where the factors reflect the abundance of C and O in each functional group. The data of each pressure series are then again normalized to unity for a data point taken at the beginning of the oxidation reaction, after about 4 min, just like in the case of the total C data in Figure S12a.

The results are shown in Figure S12b. For the reaction at 0.013 mbar and 0.04 mbar O_2 a slight increase in the total amount of C+O is observed at the beginning of the oxidation, but this increase is in the range of the experimental error. A clear increase in the carbonaceous layer, as previously observed for the oxidation of coronene[11] is thus not present for the oxidation of the oleic acid layer, where volatilization of the ligand layer dominates over the addition of material through functionalization.

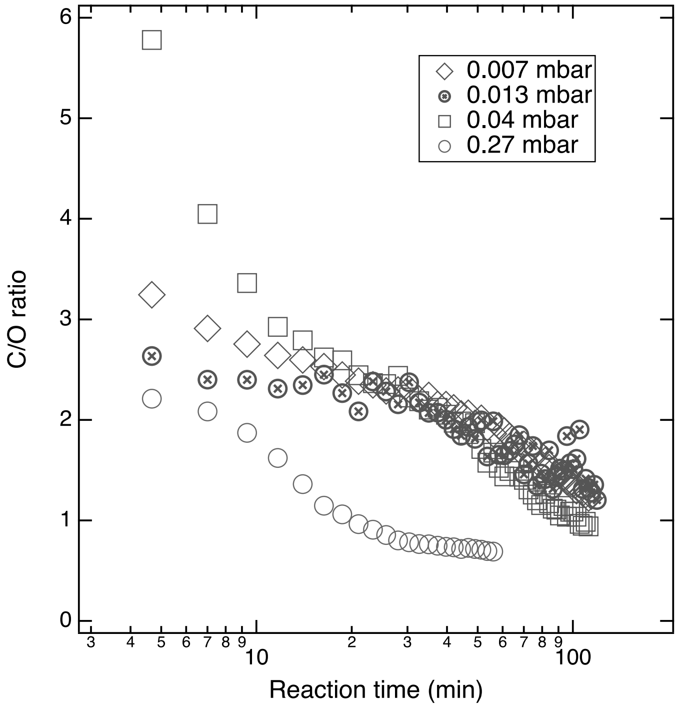

Figure S13. C/O ratio as a function of reaction time, deduced from the C 1s signal.

Another metric that can be extracted from the C 1s data is the C-to-O ratio as a function of reaction time, which informs on the degree of functionalization. This ratio is shown in Fig. S13 for the four different oxidation experiments. For the experiment at the highest O_2 pressure (0.27 mbar), where the reaction proceeds fastest, a C-to-O ratio close to unity is reached already after 30 min reaction time, while for the other O_2 pressures this value is approached but not quite reached within the duration of the oxidation of about 120 min. A C-to-O ratio close to unity is consistent with an average final reaction product similar to acetic acid.

References

(1) https://henke.lbl.gov/optical_constants/getdb2.html

(2) Mackenzie, L.E., Goode, J.A., Vakurov, A. et al., The theoretical molecular weight of $NaYF_4$:RE upconversion nanoparticles, *Sci Rep.* **2018**, *8*, 1106.

(3) Guinier, A., Fournet, G. Small-Angle Scattering of X-Rays. John Wiley and Sons: New York, 1955.

[4) Teixeira, J. Small-angle scattering by fractal systems. *J. Appl. Cryst.* **1988**, *21*, 781-785.

(5) Matsuoka, H.; Tanaka, H.; Hashimoto, T.; Ise, N. Elastic scattering from cubic lattice systems with paracrystalline distortion. *Physical Review B* **1987**, *36*, 1754-1765.

(6) Matsuoka, H.; Tanaka, H.; Hashimoto, T.; Ise, N. Elastic scattering from cubic lattice systems with paracrystalline distortion. II, *Physical Review B* **1990**, *41*, 3854-3856.

(7) https://pubchem.ncbi.nlm.nih.gov/compound/Oleic-Acid.

(8) Hopkins, J.B. BioXTAS RAW 2: New developments for a free open-source program for small-angle scattering data reduction and analysis. *J. Appl. Cryst.* **2024**, *57*, 194-208 (2024).

(9) Grant, T.D. Ab initio electron density determination directly from solution scattering data. *Nature Methods* **2018**, *15*, 191–193.

(10) The PyMOL Molecular Graphics System, Version 3.0 Schrödinger, LLC.

(11) Mysak, E.R.; Smith, J.D.; Newberg, J.T.; Ashby, P.D.; Wilson, K.R.; Bluhm, H. Competitive Reaction Pathways for Functionalization and Volatilization in the Heterogeneous Oxidation of Coronene Thin Films by Hydroxyl Radicals and Ozone. *Phys. Chem. Chem. Phys.* **2011**, *13*, 7554-7564 (2011).
